# Strip rotary tillage with a two-year subsoiling interval enhances root growth and yield in wheat

**DOI:** 10.1038/s41598-019-48159-4

**Published:** 2019-08-12

**Authors:** Jianning He, Yu Shi, Junye Zhao, Zhenwen Yu

**Affiliations:** 1 National Key Laboratory of Crop Biology, Agronomy College of Shandong Agricultural University, Tai’an, Shandong P.R. China; 2 Agricultural Information Institute of Chinese Academy of Agricultural Sciences, Beijing, P.R. China

**Keywords:** Plant physiology, Plant development

## Abstract

Excessive tillage and soil compaction threaten the sustainable farmlands in the Huang-Huai-Hai Plains of China. Our study explores tillage practices to improve soil and root ecology and promote productivity in the winter wheat fields. We tested the impact of plowing, rotary, strip rotary tillage and strip rotary tillage with a two-year subsoiling interval (SRS) on wheat yield and root quality. SRS decreased soil bulk density compared with other treatments, resulting in lower soil penetration resistance. Root morphology and weight density decreased with the increased soil depth and was higher in SRS. Moreover, SRS increased the indoleacetic acid and trans zeatin riboside levels corresponding to greater TTC reduction activities, the total and active absorption root area. SRS increased the superoxide dismutase and catalase activities and soluble protein concentration and decreased the malondialdehyde concentration. The first two factors extracted using 11 root attributes in various soil layers through principal component analysis were selected as the integrated indicators for the minimum data set, and their integrated score was calculated to quantify the root quality. Our study suggests that SRS could significantly improve root morphology and enhance the root activity in subsoil layers, thus, delaying root senescence and increasing winter wheat yield.

## Introduction

Winter wheat (*Triticum aestivum* L.), sown in the Huang-Huai-Hai Plain (3HP), the largest, highly productive plain in China^1^. However, the structural stability and nutrient content of the soil in this region has been destroyed by intensive and continuous conventional tillage practices resulting in reduced crop productivity^2,3^. Before 1990s, plowing tillage increases soil bulk density (BD) and decreases energy efficiency and economics, thus, reducing water storage capacity and nitrogen (N) accumulation^4^. After 1990s, due to its cost-effectiveness, rotary tillage has gradually replaced plowing tillage, thus, changing the working depth from 25 cm to 15 cm^5^. The subsoil compaction changes due to rotary tillage and tractor wheel during the primary, 15-cm-deep rotary tillage operation and subsequent traffic by seeding, harvesting and spreading of chemical or fertilizer operations and its remediation is more complex and costly than topsoil^6^. The decreased moisture absorption ability of the soil, as a result of the plow-pan, curbs wheat root proliferation, thus, decreasing yield and threatening the sustainable agriculture in 3HP^7,8^. Thus, tillage practices should be optimized to restore farmland ecosystems and implement sustainable cultivation practices.

The morphological and physiological properties of roots directly affect the growth and development of the above-ground plant parts, absorption of moisture and nutrients, and crop yield^9–11^. Root morphology directly influences the functionality of the root system, longer roots increase moisture absorption and nutrient supply to the plant compared with shorter roots^12^. Meanwhile, the root is also an important biosynthetic location for various hormones, organic acids and amino acids^13^. The potential for plants to obtain moisture and mineral nutrients from the soil is depends on their ability to develop extensive root systems. However, soil compaction, particularly in subsoil layers, limits the growth of deep roots, and thus, restricts the utilization of water and nutrients by the plants in the subsoil^14^. Low moisture and nutrient absorption capacity of roots in the compacted soil limits the elongation capacity of roots, which is completely curbed at the soil strength of 2MPa^15^. Increased root penetration and reduced movement of water in the soil reduces the water and nutrient availability to crops and changes the distribution of roots between soil layers and may confine root development to the upper part of the soil profile, which restricts plant availability to water and minerals^16,17^. Therefore, increased soil compaction has recently been identified as the primary limiting factor in root growth and uptake of moisture and nutrients.

Tillage affects root growth in the subsoil and its intensity and frequency can positively influence crop root growth and yield^18,19^. However, frequent tillage creates hardpans in the subsoil, which can be detrimental to root proliferation below the plow layers^20^. Reduced tillage or no tillage decreases soil disturbance and improves aggregate stability; these practices had higher root length density (RLD), root surface area density (RSD) and root weight density (RWD) than those under rotary and plowing tillage^21,22^. Elimination of tillage results in better water conservation and root system development and greater RLD compared with conventional tillage, which benefits wheat nitrogen uptake and grain yield (GY)^23^. However, Li *et al*. discovered that no tillage decreased root biomass by 26% compared with plowing tillage, mainly by reducing the primary and secondary roots^24^. Plowing tillage and rotary tillage significantly decrease BD within 0–20 cm and penetration resistance (PR) in 0–30 cm and increases the root biomass across 0–40 cm soil profile compared with no tillage^14^. As with impact of tillage on root distribution, no tillage can gradually increase mechanical impedance of the surface soil, limiting the distribution of roots in the upper soil profile and downward root progression^25^. Subsoiling loosens the hardpan and breaks up deep, compacted soil layers without bringing the infertile subsoil to the top layer; and the effects last for up to four years^26^. Others suggest that the physical effects of subsoiling do not usually last more than 1–2 years, after which the plow pan reappeared^27^. Our previous study has demonstrated that rotary tillage after subsoiling with an interval of two years contributed to enhancing GY and water use efficiency (WUE)^2,28,29^. However, studies on interval subsoiling tillage regime have focused mainly on the soil moisture content, nitrogen accumulation and translocation and the accumulation of dry matter under irrigated conditions in the 3HP^2,28,30^.

There is a dearth of information about the best tillage regime (strip rotary tillage with interval subsoiling) to achieve the optimal yield based on an integrated root quality in various soil layers. Hence, the present field study was conducted to investigate the effects of tillage regime on root ecology and sustainable productivity. We further compared conventional tillage practices with strip rotary tillage with a two-year subsoiling interval and determined the root quality based on 11 root attributes. Moreover, we determined the responses of root quality index and wheat yield to various tillage practices, examined the effects of various tillage practices on soil structure and root characters within the 0–45 cm layers, and established the minimum data set and integrated index to quantify root quality through principal component analysis (PCA).

## Results

### Soil bulk density and porosity

There were significant interactive effects in tillage practices × soil layers on BD and SPY at jointing, anthesis and 20 DAA (Table S1). ANOVA showed that tillage practices significantly affected soil bulk density (BD) and soil porosity (SPY) as influenced by varying by soil layers. In the 0–15 cm depth, the highest BD and lowest SPY (mean values of 1.52 g cm^−3^ [BD], and 42.82% [SPY]) were in SR at jointing, anthesis, and 20 DAA. In the 15–30 cm depth, R and SR resulted in higher BD and lower SPY than plowing tillage (P) and strip rotary tillage with a two-year subsoiling interval (SRS). Average BD over the three stages of SRS decreased by 5.96% and 8.39% compared with R and SR. The average SPY of SRS increased by 9.89% and 10.58%. However, at the 30–45 cm depth, the lowest BD and highest SPY were obtained with SRS, but no significant difference was observed among P, R, and SR. Significant BD reduction and SPY increase were observed under SRS because of subsoiling in the 30–45 cm layer.

### Soil penetration resistance

The average PR of the 0–15 cm soil layer in SR was 1277 kPa, higher than that in P (822 kPa), R (1003 kPa) and SRS (788 kPa) at jointing, anthesis and 20 DAA (Fig. S1). At 15–30 cm depth, R and SR resulted in higher PR than P and SRS. However, at the depth of 30–45 cm, SRS treatment had the lowest PR, whereas the values for P, R and SR treatments did not differ. Notably, the levels of PR under subsoiling reported were lower than other tillage practices, which could be attributed to the reduction in BD and the increase in SPY.

### Root weight density

**Figure 1:**
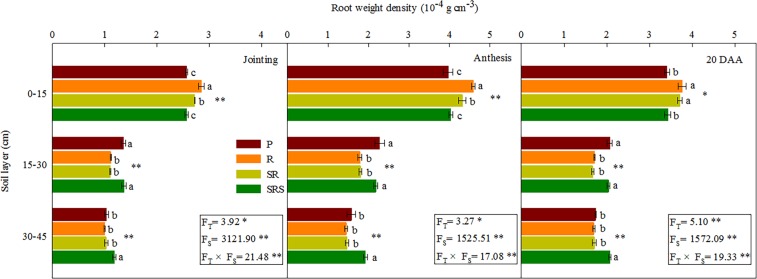
Root weight density of winter wheat within the 0–45 cm soil depth under various tillage practices at jointing, anthesis and 20 DAA in the 2014–2016 growing seasons. Results presented as the mean of 2 years. P, plowing tillage; R, rotary tillage; SR, strip rotary tillage; SRS, strip rotary tillage after subsoiling. Error bars represent SEM; n = 3. Different letters indicate significant differences between treatments. F_T_, F_S_ and F_T_ × F_S_ represent F-values of tillage, soil layers and their interaction in variance analysis respectively. *P < 0.05; **P < 0.01.

The tillage practices had a striking effect on the RWD and the effect varied based on soil layers at jointing (*P* = 0.021), anthesis (*P* = 0.039) and 20 DAA (*P* = 0.007) (Fig. 1). There was a significant interaction between tillage and soil layers with the R treatment under 0–15 cm depth having significantly greater RWD (2.85 10^−4^g cm^−3^ [jointing], 4.58 10^−4^g cm^−3^ [anthesis], 3.77 10^−4^g cm^−3^ [20 DAA]) compared with other treatments. SR had the second greatest values; SRS and P had the lowest. However, RWD did not differ between SRS and P but was significantly higher in SRS than in SR and R at a depth of 15–30 cm. Moreover, at 30–45 cm, the highest RWD was obtained in response to SRS treatment, and no significant difference was observed among P, R and SR treatments at jointing, anthesis, and 20 DAA. From the root weight density perspective, the BD was significantly and negatively correlated with the RWD in the 0–45 cm soil layers under different tillage practices (Table S2).

### Root morphology

Tillage practices, soil layers, and their interactions significantly affected the root morphology at jointing, anthesis and 20 DAA (Table 1). The RLD, RVD, and RSD of the 0–15 cm soil layer were the highest at anthesis in all treatments. The root system of wheat gradually died and decreased obviously at 20 DAA during grain filling. The RLD, RVD, and RSD increased with the filling process in the 15–45 cm soil layers, particularly in the 30–45 cm soil layer. In the 0–15 cm soil layer, the RLD, RVD, and RSD did not differ between R and SR and was higher than that for P and SRS at jointing and anthesis. At 20 DAA, in the 0–15 cm soil layer, P treatment had the lowest RLD, RVD, and RSD, whereas the values for R, SR, and SRS did not differ. In the 15–30 cm soil layer, the root morphological indices of P and SRS were higher than those of R and SR at three growth stages. However, in the 30–45 cm, the highest RLD, RVD and RSD were in the SRS treatment at jointing, anthesis, and 20 DAA. Moreover, the BD was significantly negatively correlated with the RLD, RVD, and RSD in the 0–45 cm soil layers under different tillage practices (Table S2).

**Table 1:** Root length, volume and surface area density as affected by tillage and soil layers at jointing, anthesis and 20 DAA in the 2014–2016 growing seasons.

Soil Layers (cm)	Treatment^a^	Root length density (cm cm^−3^)			Root volume density (10^−3^ cm^3^ cm^−3^)			Root surface area density (mm^2^ cm^−3^)		
		Jointing	Anthesis	20 DAA	Jointing	Anthesis	20 DAA	Jointing	Anthesis	20 DAA
0–15	P	0.86 b^b^	1.63 b	1.27 b	4.25 c	8.89 b	6.08 b	5.63 b	10.87 b	8.60 b
	R	1.00 a	1.79 a	1.39 a	4.79 a	9.51 a	6.55 a	6.19 a	11.76 a	9.29 a
	SR	0.93 ab	1.79 a	1.39 a	4.54 b	9.28 a	6.53 a	5.89 ab	11.43 ab	9.18 a
	SRS	0.86 b	1.69 b	1.37 a	4.30 bc	8.79 b	6.47 a	5.56 b	10.89 b	9.14 a
15–30	P	0.63 a	1.42 a	1.16 a	2.33 a	4.69 a	4.30 a	3.10 a	6.41 a	5.83 a
	R	0.51 b	1.25 b	1.02 b	1.86 b	3.57 b	3.65 b	2.51 b	5.50 b	5.04 b
	SR	0.52 b	1.24 b	0.99 b	1.86 b	3.76 b	3.69 b	2.50 b	5.45 b	5.05 b
	SRS	0.63 a	1.41 a	1.12 a	2.32 a	4.61 a	4.36 a	3.14 a	6.46 a	5.90 a
30–45	P	0.41 b	0.76 b	0.91 b	1.16 b	2.43 b	2.58 b	1.49 b	2.22 b	2.27 b
	R	0.38 b	0.73 b	0.88 b	1.08 b	2.42 b	2.56 b	1.43 b	2.18 b	2.21 b
	SR	0.38 b	0.73 b	0.87 b	1.09 b	2.42 b	2.57 b	1.42 b	2.12 b	2.19 b
	SRS	0.48 a	0.92 a	1.13 a	1.30 a	2.93 a	2.94 a	1.71 a	2.73 a	2.73 a
**ANOVA table (LSD protected**, ***P*** ≤ **0.05)**^**c**^										
F_T_		3.39*	12.17**	13.73**	3.64*	5.28**	12.16**	3.10*	3.11*	9.73**
F_S_		821.01**	2313.43**	260.21**	4164.96**	4182.07**	2301.90**	2854.01**	3915.71**	3467.75**
F_T_ × F_S_		15.10**	24.70**	11.38**	23.75**	20.00**	12.22**	15.15**	12.39**	9.92**
CV		0.35	0.31	0.17	0.56	0.54	0.37	0.55	0.58	0.50

^a^P, plowing tillage; R, rotary tillage; SR, strip rotary tillage; SRS, strip rotary tillage after subsoiling. ^b^Results presented as the mean of 2 years; Values followed by different letters within the same column are significantly different at *P* < 0.05. ^c^F_T_, F_S,_ and F_T × _F_S_ represent F-values of tillage, soil layers and their interaction in variance analysis respectively; *P < 0.05; **P < 0.01; CV, coefficient of variation.

### Root hormones

**Figure 2:**
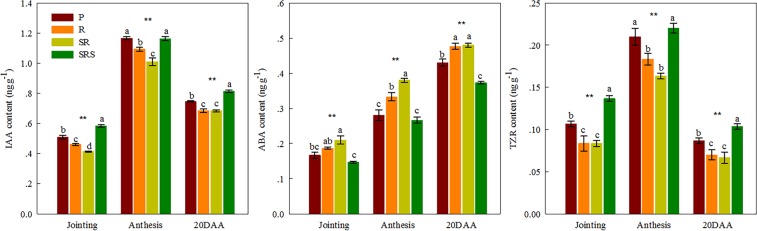
Indoleacetic acid, abscisic acid and trans zeatin riboside content in winter wheat root under various tillage practices at jointing, anthesis and 20 DAA practices in the 2014–2016 growing seasons. Results presented as the mean of 2 years. P, plowing tillage; R, rotary tillage; SR, strip rotary tillage; SRS, strip rotary tillage after subsoiling. Error bars represent SEM; n = 3. Different letters indicate significant differences between treatments. *P < 0.05; **P < 0.01.

At jointing, SRS resulted in the highest IAA and TZR content followed by P values with the lowest for R and SR (Fig. 2). P, R, and SR relatively decreased the IAA levels (12.07%, 20.69%, and 29.31%, respectively) and TZA content (21.43%, 42.85%, and 42.86%, respectively) compared with SRS. However, the lowest ABA content was obtained in response to SRS treatment. At anthesis, the IAA and TZR content did not differ between P and SRS and were higher than that for R and SR; SR showed the lowest values. However, ABA content for SR was higher than that for P, R, and SRS. At 20 DAA, the IAA and TZR content in SRS were the highest, followed by P; the lowest values were obtained in R and SR. The ABA content in R and SR were higher than that in P and SRS, and the values in P were higher than those in SRS. Moreover, the BD was significantly and negatively correlated with the IAA and TZR content but was not correlated to the ABA (*P* = 0.236) content (Table S2).

### Root TTC reduction activities and absorption area

**Figure 3:**
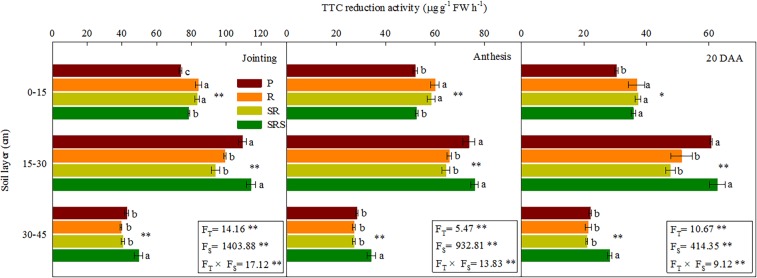
Root TTC reduction activities of winter wheat within the 0–45 cm soil depth under various tillage practices at jointing, anthesis and 20 DAA in the 2015–2016 growing seasons. P, plowing tillage; R, rotary tillage; SR, strip rotary tillage; SRS, strip rotary tillage after subsoiling. Error bars represent SEM; n = 3. Different letters indicate significant differences between treatments. F_T,_ F_S_ and F_T_ × F_S_ represent F-values of tillage, soil layers and their interaction in variance analysis respectively. *P < 0.05; **P < 0.01.

The tillage practices had a substantial effect on RTR, RTA, and RAA with the magnitude of the effect varying by soil layers at jointing, anthesis and 20 DAA (Fig. 3 and Table 2). There was a significant interaction between tillage and soil layers with the R and SR in the 0–15 cm layer having greater RTR, RTA and RAA compared with P and SRS at jointing and anthesis. However, at 20 DAA, root TTC reduction activities (RTR), root total absorption area (RTA), and root active absorption area (RAA) did not differ between R, SR and SRS but were higher than those in response to P. In the 15–30 cm layer, RTR, RTA, and RAA did not differ between SRS and P but was higher in SRS than in R and SR at three growth stages. In the 30–45 cm layer, SRS had the highest RTR, RTA and RAA, whereas the RTR, RTA and RAA values for P, R and SR did not differ at jointing, anthesis, and 20 DAA. Moreover, the soil layers had a significant effect on RTA and RAA with the superficial layer having greater levels compared with the 15–45 cm layer. However, the highest RTR was obtained for the 15–30 cm soil layer. As shown in Table S2, the BD was significantly, negatively correlated with the RTR, RTA and RAA in the 0–45 cm soil layers under different tillage practices.

**Table 2:** Root total and active absorption area as affected by tillage and soil layers at jointing, anthesis and 20 DAA in the 2014-2016 growing seasons.

Soil Layers (cm)	Treatment^a^	Root total absorption area (m^2^ m^−3^)			Root active absorption area (m^2^ m^−3^)		
		Jointing	Anthesis	20 DAA	Jointing	Anthesis	20 DAA
0–15	P	40.43 b^b^	35.74 b	27.52 a	20.29 b	17.96 b	11.71 b
	R	42.37 a	38.13 a	28.03 a	22.54 a	19.54 a	13.19 a
	SR	42.30 a	37.98 a	27.88 a	22.01 a	19.24 a	13.13 a
	SRS	39.98 b	35.98 b	27.49 a	20.20 b	18.10 b	13.10 a
15–30	P	21.72 a	18.48 a	14.91 a	12.67 a	10.01 a	7.75 a
	R	19.39 b	15.99 b	12.79 b	10.55 b	7.62 b	6.33 b
	SR	19.12 b	15.94 b	12.89 b	10.34 b	6.99 b	6.34 b
	SRS	22.08 a	18.42 a	14.92 a	12.90 a	9.58 a	7.75 a
30–45	P	12.10 b	8.40 b	6.07 b	5.57 b	4.00 b	3.41 b
	R	11.82 b	7.93 b	5.82 b	5.21 b	4.01 b	3.19 b
	SR	11.70 b	7.90 b	5.80 b	5.06 b	4.11 b	3.23 b
	SRS	13.87 a	10.07 a	7.66 a	6.67 a	5.12 a	4.40 a
**ANOVA table (LSD protected**, ***P*** ≤ **0.05)**^**c**^							
FT		3.23*	3.14*	9.05**	4.58*	4.35*	14.14**
FS		5674.01**	5543.24**	4532.40**	3606.86**	2570.46**	2360.58**
FT × FS		14.18**	13.86**	6.58**	25.20**	15.99**	13.37**
CV		0.50	0.58	0.57	0.52	0.59	0.50

^a^P, plowing tillage; R, rotary tillage; SR, strip rotary tillage; SRS, strip rotary tillage after subsoiling. ^b^Results presented as the mean of 2 years; Values followed by different letters within the same column are significantly different at *P* < 0.05. ^c^ F_T_, F_S,_ and F_T_ × F_S_ represent F-values of tillage, soil layers and their interaction in variance analysis respectively; *P < 0.05; **P < 0.01; CV, coefficient of variation.

### Root senescence

**Figure 4:**
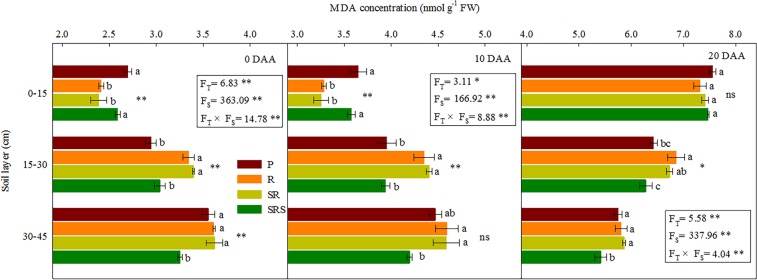
Malondialdehyde concentration in winter wheat root within the 0–45 cm soil depth under various tillage practices after anthesis in the 2014–2016 growing seasons. Results presented as the mean of 2 years. P, plowing tillage; R, rotary tillage; SR, strip rotary tillage; SRS, strip rotary tillage after subsoiling. Error bars represent SEM; n = 3. Different letters indicate significant differences between treatments. F_T_, F_S_ and F_T_ × F_S_ represent F-values of tillage, soil layers and their interaction in variance analysis respectively. *P < 0.05; **P < 0.01; ns, not significant.

The tillage practices and soil depth interacted to affect the SOD and CAT activities and SP and MDA concentration (Fig. 4 and Table 3). ANOVA showed that there were significant differences in root senescence among tillage practices and soil layers. SOD and CAT activities and SP concentrations at 0 and 10 DAA decreased with soil depth, whereas the MDA concentration increased. At 20 DAA, SOD and CAT activities and SP concentrations increased with soil depth, whereas the MDA concentration decreased. In the 0–15 cm layer, SOD and CAT activities and SP concentrations in R and SR were higher than those in P and SRS at 0 and 10 DAA. At 20 DAA, the SOD and CAT activities and SP concentrations did not differ among the R, SR, and SRS and were higher than that for P. MDA concentration of root did not differ between P and SRS and was higher than that in R and SR at 0 and 10 DAA. At 20 DAA, the MDA concentration did not differ in all treatments. In the 15–30 cm layer, the SOD and CAT activities and SP concentrations of P and SRS were higher than those of R and SR at 0 DAA, 10 DAA and 20 DAA. However, R and SR had the highest MDA concentration. In the 30–45 cm layer, SRS had the highest SOD and CAT activities and SP concentrations and the lowest MDA concentration during grain filling. Moreover, the BD was markedly and negatively correlated with the SOD and CAT activities and SP concentrations; however, a significant and positive relationship was found between BD and MDA concentration (Table S2).

**Table 3:** Superoxide dismutase, catalase activities and soluble protein concentration in winter wheat root as affected by tillage and soil layers after anthesis in the 2014–2016 growing seasons.

Soil Layers (cm)	Treatment^a^	SOD activity (Ug^−1^ FW)			CAT activity (mmol H_2_O_2_ g^−1^ FW)			SP concentration (mg g^−1^ FW)		
		0 DAA	10 DAA	20 DAA	0 DAA	10 DAA	20 DAA	0 DAA	10 DAA	20 DAA
0–15	P	243.50 c^b^	210.01 b	64.26 b	4.63 b	3.69 b	1.62 b	18.75 c	16.74 b	6.84 b
	R	271.18 a	231.58 a	71.05 a	4.87 a	3.97 a	1.78 a	20.90 a	18.89 a	7.71 a
	SR	256.24 b	225.17 a	70.88 a	4.85 a	3.92 a	1.75 a	19.65 b	18.46 a	7.49 a
	SRS	239.71 c	211.26 b	70.64 a	4.64 b	3.69 b	1.73 ab	18.68 c	16.71 b	7.44 a
15–30	P	208.16 a	185.83 a	101.47 a	4.03 a	3.11 a	2.26 a	16.93 a	14.97 a	9.32 a
	R	188.79 b	163.23 b	90.18 b	3.68 b	2.74 b	2.09 b	15.00 b	13.04 b	8.44 b
	SR	187.50 b	160.56 b	88.52 b	3.61 b	2.72 b	2.06 b	14.82 b	12.82 b	8.38 b
	SRS	204.74 a	185.20 a	98.45 a	3.99 a	3.10 a	2.23 a	16.59 a	14.61 a	9.23 a
30–45	P	173.48 b	155.15 b	108.45 b	3.51 b	2.57 b	2.41 b	12.39 b	10.39 b	9.66 b
	R	167.69 b	151.35 b	106.99 b	3.48 b	2.51 b	2.37 b	11.95 b	9.97 b	9.53 b
	SR	168.85 b	151.52 b	106.80 b	3.42 b	2.50 b	2.34 b	12.02 b	10.02 b	9.50 b
	SRS	190.19 a	176.89 a	128.34 a	3.76 a	2.81 a	2.67 a	14.09 a	12.06 a	10.72 a
**ANOVA table (LSD protected**, ***P*** ≤ **0.05)**^**c**^										
F_T_		3.12*	7.52**	10.49**	4.08*	4.74**	14.43**	14.74**	5.66**	10.05**
F_S_		707.46**	383.15**	291.13**	403.28**	535.93**	533.55**	1513.40**	1120.02**	227.80**
F_T_ × F_S_		21.94**	17.68**	7.01**	8.68**	12.79**	11.08**	44.33**	35.38**	8.36**
CV		0.17	0.16	0.21	0.14	0.18	0.15	0.19	0.22	0.13

^a^ P, plowing tillage; R, rotary tillage; SR, strip rotary tillage; SRS, strip rotary tillage after subsoiling. ^b^Results presented as the mean of 2 years; Values followed by different letters within the same column are significantly different at *P* < 0.05. ^c^ F_T_, F_S,_ and F_T_ × F_S_ represent F-values of tillage, soil layers and their interaction in variance analysis respectively; *P < 0.05; **P < 0.01; CV, coefficient of variation.

### Grain yield

**Figure 5:**
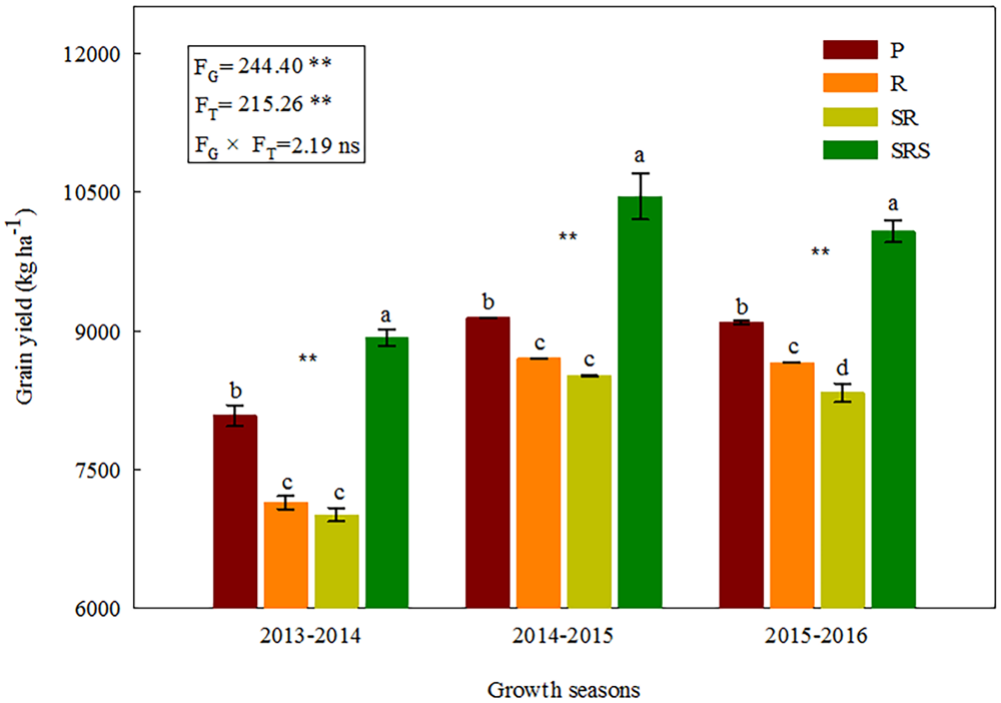
Grain yield under various tillage practices in the 2013–2016 growing seasons. P, plowing tillage; R, rotary tillage; SR, strip rotary tillage; SRS, strip rotary tillage after subsoiling. Error bars represent SEM; n = 3. Different letters indicate significant differences between treatments. F_G_, F_T_ and F_G × _F_T_ represent F-values of growth seasons, tillage and their interaction in variance analysis respectively. *P < 0.05; **P < 0.01; ns, not significant.

Tillage and growth seasons significantly affected GY, but there were no significant interaction effects (*P* = 0.079) (Fig. 5). The highest GY (mean values of 9816 kg ha^−1^) were observed in response to the SRS followed by P and the lowest with R and SR. Compared with SRS, the mean value of GY was lower by 10.67% (P), 16.82% (R) and 19.03% (SR). The average EB was highest in SRS at 1,247 US$ ha^−1^ (Fig. S2).

### Evaluation of root traits and association with grain yield

The variance was homogeneous at *P* < 0.05 (Table S4). All root attributes were significantly negatively related to MDA, and positively related to RWD (Table 4). Positive correlations were observed in root morphology (RLD, RVD, and RSD) and root activity (RTR, RTA, and RAA). Root activity strongly influenced root senescence (SOD, CAT, and SP), and showed a positive correlation.

**Table 4:** The correlation coefficient showing the interactions among different root attributes (n = 9).

	RWD	RLD	RVD	RSD	RTR	RTA	RAA	MDA	SOD	CAT	SP
RWD^a^	1.000										
RLD	0.886**^b^	1.000									
RVD	0.987**	0.924**	1.000								
RSD	0.953**	0.973**	0.982**	1.000							
RTR	0.333*	0.713**	0.409**	0.557**	1.000						
RTA	0.978**	0.943**	0.994**	0.990**	0.458**	1.000					
RAA	0.981**	0.942**	0.996**	0.988**	0.458**	0.994**	1.000				
MDA	−0.950**	−0.929**	−0.947**	−0.939**	−0.504**	−0.945**	−0.954**	1.000			
SOD	0.969**	0.928**	0.972**	0.964**	0.463**	0.965**	0.974**	−0.953**	1.000		
CAT	0.976**	0.909**	0.974**	0.951**	0.415**	0.966**	0.974**	−0.967**	0.966**	1.000	
SP	0.932**	0.976**	0.947**	0.970**	0.627**	0.954**	0.960**	−0.964**	0.970**	0.947**	1.000

^a^RWD, root weight density; RLD, root length density; RVD, root volume density; RSD, root surface area density; RTR, root TTC reduction activities; RTA, root total absorption area; RAA, root active absorption area; MDA, malondialdehyde; SOD, superoxide dismutase; CAT, catalase; SP, soluble protein. ^b^*P < 0.05; **P < 0.01.

PCA analysis showed that the root attributes differed remarkably with changing tillage practices. From the PCA of tillage practices (KMO = 0.921), we identified two principal components (PC), each with an eigenvalue greater than 1 (Table 5). The two PCs (PC1 and PC2) explained 98.20% of the total variability. The eigenvalue for PC1 was 8.70, which explained 79.10% of the variability. The PC1 was a contrast of RWD, RLD, RVD, RSD, RTA, RAA, SOD, CAT and SP with positive loadings against the negative loadings of MDA. PC2 explained 19.10% of the variability (eigenvalue = 2.10) and consisted of only positive loadings for RTR.

**Table 5:** Variable loading coefficients (eigenvectors) of the first two factors extracted using 11 root attributes, their eigenvalues, and individual and cumulative percentage of total variance explained by each factor.

Root attributes^a^	Component		Commonalities^b^
	1	2	
RWD	**0.987**	0.140	0.993
RLD	**0.820**	0.565	0.992
RVD	**0.971**	0.222	0.991
RSD	**0.913**	0.385	0.983
RTR	0.197	**0.979**	0.998
RTA	**0.953**	0.276	0.984
RAA	**0.957**	0.274	0.992
MDA	**−0.915**	−0.330	0.946
SOD	**0.943**	0.284	0.969
CAT	**0.959**	0.227	0.971
SP	**0.877**	0.465	0.984
Eigenvalue	8.699	2.104	
Individual variance	0.791	0.191	
Cumulative variance	0.791	0.982	

^a^RWD, root weight density; RLD, root length density; RVD, root volume density; RSD, root surface area density; RTR, root TTC reduction activities; RTA, root total absorption area; RAA, root active absorption area; MDA, malondialdehyde; SOD, superoxide dismutase; CAT, catalase; SP, soluble protein.^b^The commonality of a variable represents the amount of variance in the variable that is accounted for. The larger the commonality for each variable, the more accurate the factor analysis model.

The score values of integrated indicators in the minimum data set were calculated to quantify root quality under various tillage practices (Table 6). The score of the first and second factor and the integrated score were significantly affected by tillage practices, soil layers, and their interactions indicating their effect on root quality. The score of the first and second factor did not differ between R and SR and was higher than that for P and SRS in 0–15 cm. At 15–30 cm, the higher score of the first and second factor were observed under P and SRS than under R and SR. At 30–45 cm, the SRS had the highest score of the first and second factor. Like the score of various factors, the same trend was observed for the integrated score with changing tillage practices, which was determined as the most significant integrated score under SRS treatment. Moreover, as shown in Table S5, the BD was significantly and negatively correlated with the integrated score for root quality; and correlation analysis further showed that the GY was distinctly and positively correlated to the integrated score for root quality under various tillage practices.

**Table 6:** Treatment means and analysis of variance for scores of the first two factors extracted using 11 root attributes, and their integrated score for root quality under different tillage.

Soil Layers (cm)	Treatment^a^	Score		Integrated score
		Factor 1	Factor 2	
0–15	P	1.23 b^b^	−0.24 b	0.93 c
	R	1.57 a	0.15 a	1.27 a
	SR	1.45 a	0.10 a	1.17 b
	SRS	1.25 b	−0.18 b	0.95 c
15–30	P	−0.45 a	1.37 a	−0.10 a
	R	−0.84 b	1.00 b	−0.47 b
	SR	−0.86 b	0.92 b	−0.50 b
	SRS	−0.57 a	1.50 a	−0.16 a
30–45	P	−0.73 b	−1.21 b	−0.81 b
	R	−0.78 b	−1.27 b	−0.86 b
	SR	−0.79 b	−1.26 b	−0.87 b
	SRS	−0.47 a	−0.88 a	−0.54 a
**ANOVA table (LSD protected**, ***P*** ≤ **0.05)**^**c**^				
F_T_		6.31**	5.36**	27.02**
F_S_		3596.96**	996.73**	8296.22**
F_T_ × F_S_		25.54**	10.74**	94.06**

^a^P, plowing tillage; R, rotary tillage; SR, strip rotary tillage; SRS, strip rotary tillage after subsoiling. ^b^Results presented as the mean of 2 years; Values followed by different letters within the same column are significantly different at *P* < 0.05. ^c^F_T_, F_S,_ and F_T_ × F_S_ represent F-values of tillage, soil layers and their interaction in variance analysis respectively; *P < 0.05; **P < 0.01.

## Discussion

### Soil compaction responses to various tillage practices

Intensive tillage adversely affected the soil structure and caused excessive decomposition of aggregates, thus, increasing the potential for soil erosion^31,32^. Compared with shallow plowing, deep plowing breaks up dense soil layers, improves the soil properties, and decreases the BD in the tilled layer, but may move the compaction problem to a deeper layer due to the effect of the plow pan below the tilled layer^6^. Rotary tillage can effectively break the argillic horizon layer and decrease soil compaction; however, it loosens the topsoil so much that it is no longer suitable for growing winter wheat^7^. He *et al*. demonstrated that eliminating tillage for long periods results in a positive effect for the first several years but long-term reductions in tillage or no tillage increase the BD and PR in the 10–20 cm soil layer^26^; thus, leading to a reduction in air-filled pore space^33^. Consequently, single continuous tillage may shift the compaction problem to a deeper layer and would not create an ideal favorable soil environment for root growth and crop production. To restore farmland ecosystems and implement sustainable tillage practices in the 3HP of China, we designed a new “combined tillage practices”—strip rotary tillage after a two-year subsoiling interval by combining advantages of strip rotary tillage and subsoiling.

Although BD and PR varied throughout the wheat growing season, the difference between tillage practices for BD and PR varied for long after tillage implementation^6^. Our study showed that the new tillage regime of SRS could result in remarkable positive shifts in the soil physical structure. Specifically, SRS could be characterized by a distinctly decreased BD and PR, increased SPY in the 15–45 cm soil depth (Fig. S1; Table S1). These results demonstrate that SRS effectively improve the soil quality by loosening the soil and eliminating soil compaction, which benefited wheat root growth and development.

### Root growth and development responses to various tillage practices

**Figure 6:**
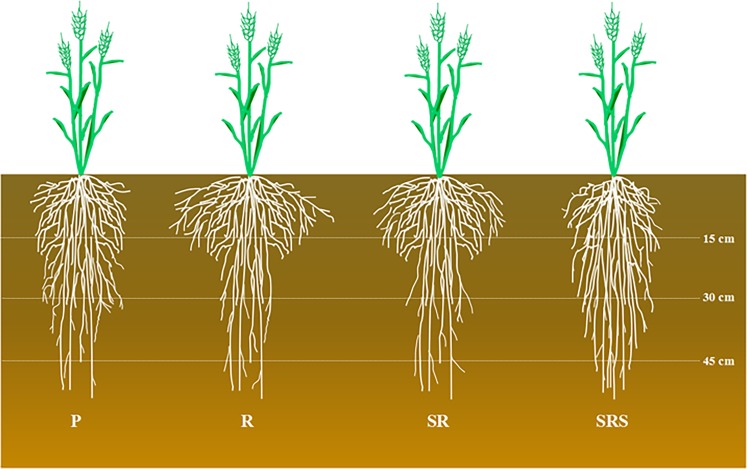
Schematic diagram of root morphology within the 0–45 cm soil depth under various tillage practices. P, plowing tillage; R, rotary tillage; SR, strip rotary tillage; SRS, strip rotary tillage after subsoiling.

A strong root system could increase root anchorage and absorptive capacity for water and nutrients, leading to high yield and resistance to root lodging^19^. Root development is known to be promoted by rotary tillage, which has higher RLD and root diameter; and the RLD under deep plowing (to a depth of 30 cm) was higher than that under no tillage^19,25^. However, there has been no systematic study of root morphological characteristics of winter wheat under SRS so far, particularly in the 3HP of China. Our study systematically explored the root morphology of winter wheat in 0–45 cm soil depth across various tillage practices and crop development stages and indicated a significant negative correlation of RWD, RLD, RVD, and RSD with BD in the 0–45 cm soil layers under various tillage practices. In this study, at jointing, anthesis, and 20 DAA, R and SR had highest RWD, RLD, RVD and RSD within the 0–15 cm soil layers. At a depth of 15–30 cm, P and SRS significantly increased the RWD, RLD, RVD, and RSD compared with R and SR; however, in 30–45 cm, SRS showed higher RWD, RLD, RVD and RSD (Fig. 6).

Root growth and spatial distribution are vital to water extraction and nutrient uptake and subsequent yield. To further explore the physiological mechanism of tillage on the root system, in the current study, we determined the detailed root growth patterns of winter wheat under various tillage practices, and the findings began to fill the gaps. Subsoiling or deep plowing provide a less restricted soil physical environment for crop root growth than conventional tillage, reduced tillage or a no tillage^6,11,34^. The RTR did not differ between plowing tillage and subsoiling and was higher than that for rotary tillage^35^. However, Li *et al*. indicated that RTA and RAA in rotary tillage were 9.60% and 3.30% higher than those in plowing tillage at 20 DAA^36^. The findings of this study consistently showed that SRS significantly increased the mean IAA and TZR contents, decreased ABA content compared to other treatments, corresponding to significantly greater RTR, RTA, and RAA at jointing anthesis and 20 DAA. Further, in the present study, SRS resulted in the highest SOD, CAT activities and SP concentration, lowest MDA concentration within the 30–45 cm, which may be due to the improved root activity. Interestingly, all indicators of root physiology except ABA content were significantly negatively correlated to BD and positively correlated to the MDA concentration (Table S2).

These results suggest that the combined tillage practices of SRS could significantly improve root morphology and physiology in the wheat growing seasons, which benefit the soil ecological environment. However, this demonstrates that the poor root system of wheat due to soil compaction under traditional tillage is the primary factor affecting yield reduction and weak root activity. Our results provide a physical and chemical mechanism for improving root morphological and physiological characters by using subsoiling combined with strip rotary tillage before seeding.

### Grain yield responses to various tillage practices

The level of both tillage management and soil productivity could be well reflected by GY. Compared with plowing tillage, reduced tillage or no tillage could increase GY between 9.0% and 37.0%^37^. A two-year experiment also showed that the no tillage increased GY by 23.50% compared with plowing tillage. However, Vita *et al*. found that GY was comparable in soils with no-tillage and plowing tillage in the first two years, but in the third year, the plowing tillage (4.6 Mg ha^−1^) had greater GY than no-tillage (2.9 Mg ha^−1^)^38^. Our results showed the mean value of GY under P increased by 10.33% compared with that under SR. This result concurred with those of Arvidsson *et al*. who found that GY will be reduced with long-term no tillage, caused by soil compaction and poor establishment due to the lack of a seedbed^39^. Considering the negative effects of repeated single tillage, a combined tillage practice study is desirable. After continuous 9 years of experiments, we found that the SRS substantially enhanced GY, WUE, and EB of winter wheat. The mean yield of SRS over three years increased by 11.94%, 20.22%, and 23.50% compared with that of P, R and SR, respectively (Fig. 5). Moreover, growth seasons and tillage practices had distinct, not cumulative effects on wheat yield. Thus, the effect of tillage on grain yields was not remarkably affected by growth seasons, and the results had good reproducibility with a consistent annual trend.

### Root traits and its association with grain yield

The root system is the basis of wheat growth, as robust roots provide adequate nutrients and water for development, which are conducive to high GY^10,11^. The root system serves as a bridge between the impacts of tillage practices on soil and changes in shoot function and harvested yield^13,34^. Soil compaction, particularly in the subsoil layers, dramatically limits deep roots growth and development, and thus restricts the utilization of moisture and nutrients by the plants in the subsoil^17^. Suitable tillage practices could improve the soil structure by loosening the soil and reversing soil compaction in deeper soil layers, thus, resulting in an improved root system, which is the most critical role that tillage plays in the soil-plant systems^18^. However, since there is no information determining the suitable tillage practice to achieve the optimal yield based on an integrated root quality in various soil depth, our study to evaluate the root quality based on 11 root attributes across various tillage practices and soil depth is of great significance.

Our study showed that all root attributes were significantly positively correlated, but negatively related to MDA (Table 4). Considering the significant interdependence between root indices, we used the first two factors (explained 98.20% of the total variability) as the integrated indices for the minimum data set to evaluate root quality (Table 5). Then, we used PCA to score root traits into quality indices that represent their root functions. Our data indicated that R had the highest the integrated score for root quality at 0–15 cm. However, a significant increase was observed in the integrated score under P and SRS at 15–45 cm, with the highest score under SRS at 30–45 cm (Table 6). These results suggested that rotary tillage and strip rotary tillage exerted an adverse effect on root quality in 15–45 cm soil layers, whereas subsoiling combined with strip rotary tillage could effectively improve the root traits.

Moreover, the BD was significantly negatively correlated with the integrated score for root quality; but a significant, positive correlation was found between the wheat yield and the combined score for root quality across all tested treatments (Table S5), which could explain the responses of GY of wheat to various tillage practices. Although R and SR could effectively promote root distribution and physiological activity in the 0–15 cm, these practices did not improve root growth and development at the whole 0–45 cm depth. Consequently, continuous rotary tillage and strip rotary tillage decreased the mean GY for the three years by 16.82% and 19.03% compared with SRS (Fig. 5). We showed that the SRS is the effective tillage practice for wheat productivity in the 3HP as it enhances root systems at different soil profile depths by improving soil environment such as soil compaction is decreased, resulting in higher GY. This study offers a basis for method development to adequately measure root morphological and physiological characteristics to reduce wheat productivity risks in the 3HP. Under the current experimental condition, SRS would be the most effective tillage practice to improve the decline of farmland sustainable productivity in the 3HP and similar areas.

## Conclusion

The present study provides an overview of the effects of various tillage practices on soil properties, root morphological distribution, and physiological activity and yields for winter wheat. “Combined tillage” (strip rotary tillage with a two-year subsoiling interval) was associated with a more favorable soil environment for wheat root growth than single tillage practices, which favors higher GY. SRS significantly improved a wide range of soil physical parameters, decreased the soil BD and PR, and maintained high soil porosity. These effects resulted in greater RWD and suitable spatial distribution, leading to higher IAA, TZR content and root activities, thus, delaying the senescence of wheat roots to achieve the highest GY.

Moreover, yield was significantly correlated with the integrated score for root quality, which explained the positive response of the yield to SRS. The positive changes in soil aeration and structure, improved root distribution, increased the root activities and delayed root senescence due to SRS likely contributed to the significantly higher winter wheat yield. Thus, the SRS may be a significant step toward sustainable productivity of farmland in the 3HP of China.

## Materials and Methods

### Experimental site

The study was initiated in 2007 in the village of Shijiawangzi, Yanzhou, Shandong Province, Northern China. This village is located in the center of the 3HP, and its environment is representative of the region. Table S6 provides information on the geography, climate and soil properties of the site. The monthly precipitation in the growing seasons of winter wheat during 2010–2016 years and mean annual precipitation during the 43 yr (1966–2010) at the experimental site are shown in Fig. S3. The local meteorological bureau of the Yanzhou district, located 0.5 km from the Shijiawangzi experimental site, provided these data.

### Experimental design

The tillage study was conducted over a period of nine years. There were four tillage treatments: plowing tillage (P), rotary tillage (R), strip rotary tillage (SR), and strip rotary tillage with a two-year subsoiling interval (SRS). In SRS, subsoiling was performed in the years 2007–2008, 2010–2011 and 2013–2014 winter wheat growing seasons (strip rotary tillage was implemented every season). The operational procedures for each tillage practice are shown in Table S7. All tillage treatments were replicated thrice and followed a randomized block design. Each plot was 40 m × 4 m.

### Crop management

The winter wheat cultivar ‘Jimai 22’ was selected because it is the most widely planted cultivar in the 3HP. In this study, data collected from 2014 to 2016 were analyzed. Seeds were sown on 8 October 2014 and 16 October 2015 and plants were harvested on 13 June 2015 and 12 June 2016, respectively. At the sowing stage, 105 kg N ha^−1^ urea, 150 kg P_2_O_5_ ha^−1^ diammonium phosphate, and 150 kg K_2_O ha^−1^ potassium chloride were surface-applied to the soil before tillage. At the jointing stage, 135 kg N ha^−1^ of urea was applied to the soil at a depth of 4 cm by ditching^40^. The other management practices such as pest control were similar to conventional practices for wheat.

### Sampling method and measurement

#### Soil bulk density and soil porosity

BD at a soil depth of 0–15, 15–30 and 30–45 cm, as well as undisturbed 100 cm^3^ core samples, were collected from three independent areas within each experimental plot at the jointing, anthesis and 20 days after anthesis (DAA). The core samples were immediately weighed, dried at 105 °C for 48 h to a constant weight and reweighed in an oven to determine the BD^41^. Soil porosity (SPY) was calculated using an equation based on the relationship between bulk and particle densities. SPY was expressed as a percentage^42^:

1 $${S}PY({ \% })=(1-\frac{{BD}}{{PD}})\times {100}$$

where *SPY* (%) is the soil porosity, *BD* (g cm^−3^) is the soil bulk density, *PD* (g cm^−3^) is the particle density.

#### Soil penetration resistance

To characterize the degree of soil loosening among the tillage systems, PR was determined down the soil profile to 45 cm, at intervals of 2.5 cm, using an electronic cone penetrometer (Model SC-900, Spectrum Technologies Inc., Chicago, IL, USA). The PR (three replicates for each experimental plot) was measured at jointing, anthesis and 20 DAA.

#### Root sampling and measurements

Roots were collected from each treatment (three replicates) and taken at jointing, anthesis, 10 DAA, and 20 DAA. As described by^43^, we carefully removed the aboveground parts before root sampling; the root samples were collected at 15 cm increments down to 45 cm. Two cores per plot were collected: one within the crop row and one midway between rows. The resultant mixture of roots and soil was then placed in a 100-mesh nylon bag and washed with tap water. The soil and roots were carefully separated and refrigerated for further testing.

The root samples were oven-dried (DHG-9420A, Bilon Instruments Co. Ltd., Shanghai, China) at 80 °C after heat processing at 105 °C for 30 min to a constant weight and then measured root weight. Root sample images were scanned using a Epson V700 scanner (Seiko Epson Corp, Japan) and analyzed with the software WinRHIZO 2013 (Regent Instruments, Canada Inc) to measure root length, root volume and root surface area, as described by Xu *et al*. and Liu *et al*.^10,11^. The root weight density (RWD, 10^−4^g cm^−3^), root length density (RLD, cm cm^−3^), root volume density (RVD, 10^−3^ cm^3^ cm^−3^) and root surface area density (RSD, mm^2^ cm^−3^) were calculated by the following formulas^40^:

2 $$RWD=M/V$$

3 $$RLD=L/V$$

4 $$RVD={V}_{R}/V$$

5 $$RSD=S/V$$

where *M* is the root weight (g), *L* is the root length (cm), *V*_*R*_ is the root volume (cm^3^), *S* is the root surface area (mm^2^), and *V* is the volume of the soil sample (cm^3^).

The root hormones were measured by high-performance liquid chromatography-mass spectrometry^44^. The root activity was determined using the TTC method^45^ and was recorded as a measure of the triphenyl tetrazolium chloride (TTC) reduction activity. The total and active absorption areas of fresh root samples were measured using the Methylene blue dyeing method^13^.

Four root senescence indices: malondialdehyde (MDA), superoxide dismutase (SOD), catalase (CAT) and soluble protein (SP) were measured as described by Guo *et al*.^46^. MDA concentrations were assayed according to Quan *et al*.^47^, SOD activity was measured spectrophotometrically according to the inhibition in the photochemical reduction of nitroblue tetrazolium; CAT activity was assayed by measuring the initial rate of H_2_O_2_ disappearance; and SP concentration was measured according to the Coomassie brilliant blue method.

#### Grain yield

GY was determined based on the 9 m^2^ harvest areas in each plot and expressed at 12.5% grain water content^30^.

### Statistical analysis

The variance of homogeneity was determined using the Levene’s test^3^ and the normality of data was evaluated by the Shapiro–Wilk’s test^48^. The soil properties, root traits, GY and WUE were statistically analyzed to test for differences among four tillage treatments, using the analysis of variance (ANOVA; α = 0.05) followed by least significant difference tests. All combined effects of growing seasons and tillage on GY and WUE were determined using two-way ANOVA. A two-way ANOVA was also conducted to examine the main effects and interactions of soil depth and tillage on root traits. The significant differences between tillage or soil layers were examined by one-way ANOVA. PCA was implemented to establish the minimum data set and integrated indices for root quality. The experimental data were analyzed through PCA using factor extraction with an eigenvalue > 1 and varimax rotation. Pearson’s correlation tests were used to examine possible relationships between soil properties, root characteristics, and GY. All analyses were conducted using SPSS 22.0 (SPSS Inc., Chicago, Illinois, USA).

## Supplementary information

Fig. S1, Fig.S2, Fig. S3, Table S1, Table S2, Table S3, Table S4, Table S5, Table S6, Table S7

## Data Availability

All data generated or analyzed during this study are included in this published article (and its Supplementary Information files).
