# Supplementary material for: Strip rotary tillage with a two-year subsoiling interval enhances root growth and yield in wheat
Source: Sci Rep. 2019 Aug 12;9:11678. doi: 10.1038/s41598-019-48159-4 (PMC6691134; doi:10.1038/s41598-019-48159-4)
Supplement: Supplementary file 1 — Fig. S1, Fig.S2, Fig. S3, Table S1, Table S2, Table S3, Table S4, Table S5, Table S6, Table S7 [file 41598_2019_48159_MOESM1_ESM.pdf]

Supplementary Materials for

Strip rotary tillage with a two-year subsoiling interval enhances root growth and yield in wheat

Jianning He [1], Yu Shi [1,*], Junye Zhao[1,2], Zhenwen Yu [1]

[1] National Key Laboratory of Crop Biology, Agronomy College of Shandong Agricultural University, Tai'an, Shandong, PR China

[2]Agricultural Information Institute of Chinese Academy of Agricultural Sciences, Beijing, PR China

[*]Corresponding author: E-mail address: shiyu@sdau.edu.cn

This file includes:

Fig. S1 to S3

Table S1 to S7

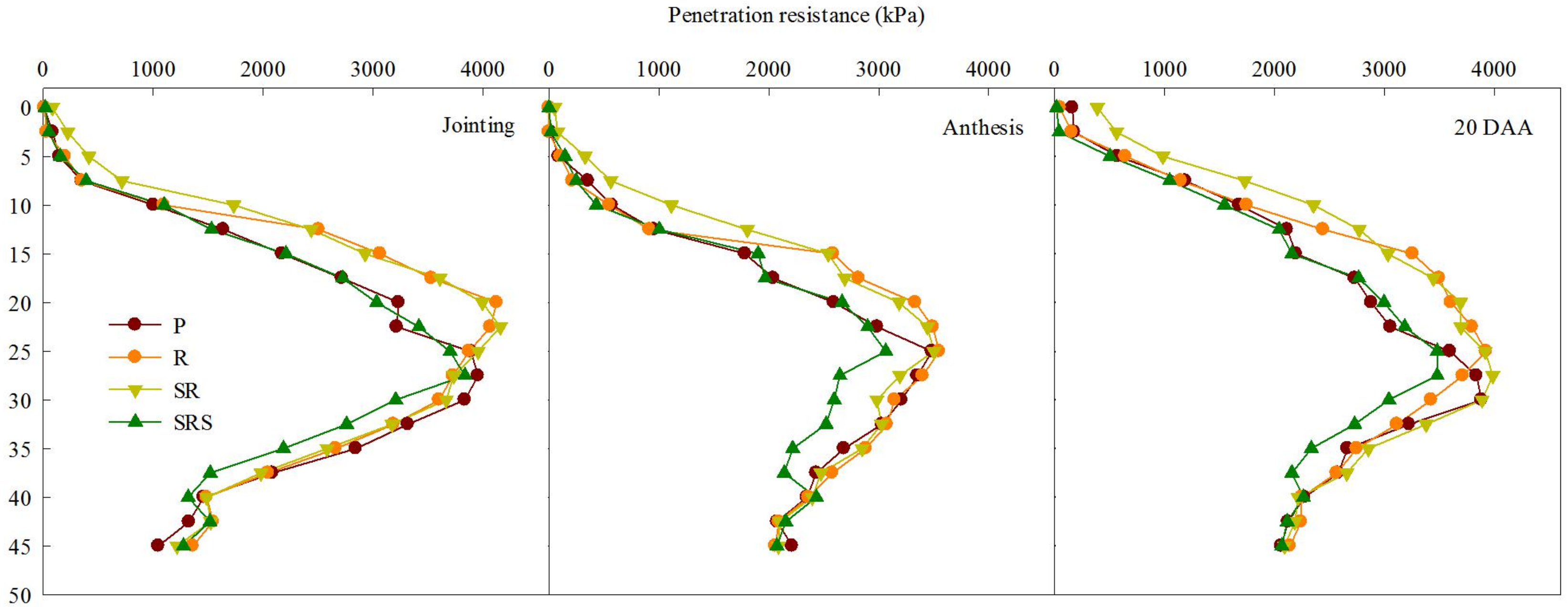

Fig. S1. Soil penetration resistance within the 0–45 cm soil depth under various tillage practices at jointing, anthesis and 20 DAA.

P, plowing tillage; R, rotary tillage; SR, strip rotary tillage; SRS, strip rotary tillage after subsoiling.

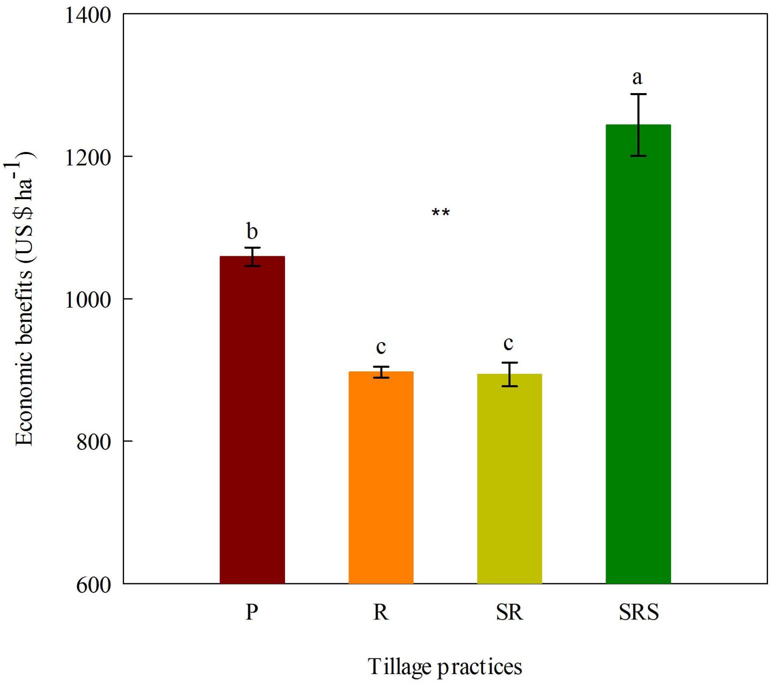

Fig. S2. Economic benefits under various tillage practices. The data were the average values in the 2013–2016 growing seasons.

P, plowing tillage; R, rotary tillage; SR, strip rotary tillage; SRS, strip rotary tillage after subsoiling.

Error bars represent SEM; n = 3. Different letters indicate significant differences between treatments. **P* < 0.05; ***P* < 0.01.

Additional information is provided in Table S3. Economic benefits = Economic outputs-Economic inputs.

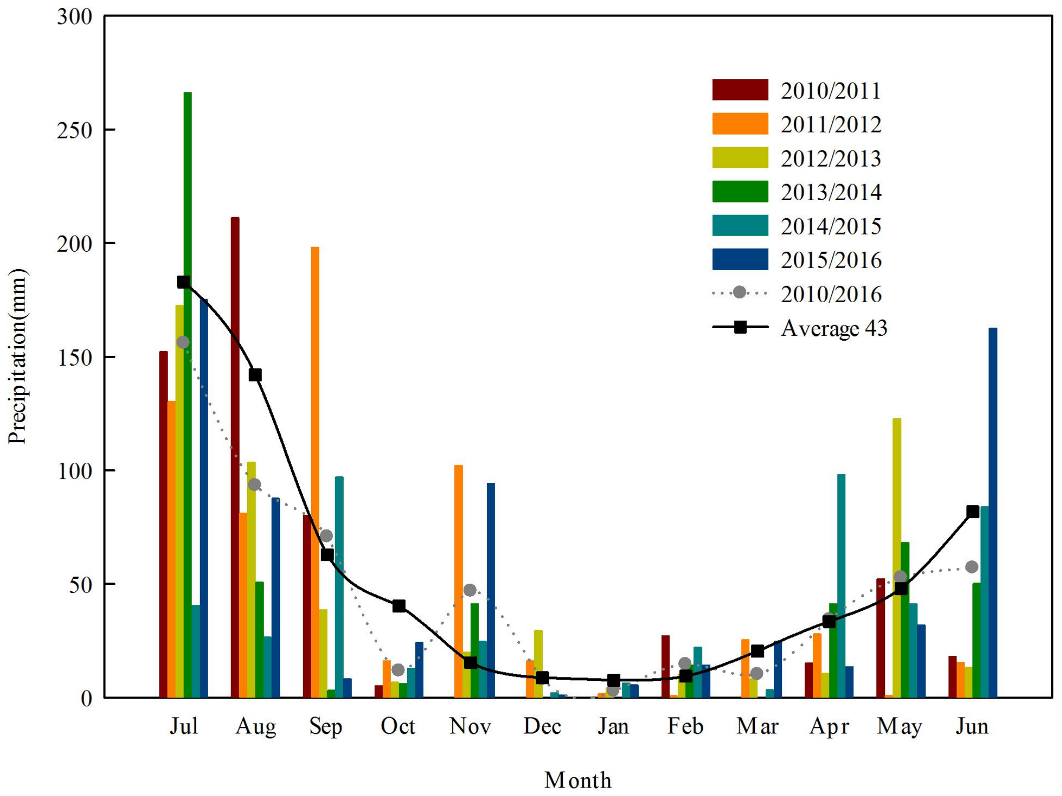

Fig. S3. The monthly precipitation in the growing seasons of winter wheat during 2010 to 2016 years and mean annual precipitation during the 43yr (1966-2010).

Table S1

Soil bulk density and porosity as affected by tillage and soil layers at jointing, anthesis and 20 DAA.

Soil Layers (cm)	Treatment[a]	Soil bulk density (g cm^{-3})			Soil porosity (%)		
		Jointing	Anthesis	20 DAA	Jointing	Anthesis	20 DAA
0-15	P	1.43b[b]	1.41b	1.42b	47.72a	47.80ab	47.28a
	R	1.43b	1.41b	1.41b	47.53a	47.54b	47.08a
	SR	1.55a	1.49a	1.53a	41.56b	43.91c	42.98b
	SRS	1.43b	1.40b	1.41b	47.99a	48.29a	47.03a
15-30	P	1.50b	1.42c	1.46b	41.90b	44.67b	43.87b
	R	1.51ab	1.49b	1.52a	41.02b	42.40c	41.29c
	SR	1.56a	1.55a	1.54a	40.98b	41.94c	41.02c
	SRS	1.45b	1.38c	1.43c	45.73a	45.94a	45.37a
30-45	P	1.55a	1.54a	1.54a	40.67b	41.95b	41.57b
	R	1.56a	1.53a	1.54a	40.81b	42.15b	41.18b
	SR	1.56a	1.55a	1.54a	40.67b	41.66b	41.48b
	SRS	1.47b	1.46b	1.46b	44.71a	45.15a	44.13a
ANOVA table (LSD protected, $P \leqslant 0.05$)[c]							
F_T		44.51**	41.28**	176.53**	52.81**	62.66**	28.21**
F_S		45.86**	52.85**	192.20**	94.47**	145.03**	69.07**
$F_T \times F_S$		6.72**	6.30**	32.51**	11.46**	8.20**	6.23**
CV		0.04	0.04	0.04	0.07	0.06	0.06

[a] P, plowing tillage; R, rotary tillage; SR, strip rotary tillage; SRS, strip rotary tillage after subsoiling. [b] Results presented as the mean of 2 years; Values followed by different letters within the same column are significantly different at $P < 0.05$. [c] F_T, F_S and $F_T \times F_S$ represent F-values of tillage, soil layers and their interaction in variance analysis respectively; $^{**}P < 0.01$; CV, coefficient of variation.

Table S2

The correlation coefficient between soil bulk density and root attributes.

	RWD	RLD	RVD	RSD	IAA	ABA	TZR	RTR	RTA	RAA	MDA	SOD	CAT	SP
BD[a]	-0.577**[b]	-0.669**	-0.590**	-0.622**	-0.498**	0.124ns	-0.526**	-0.543**	-0.589**	-0.615**	0.685**	-0.637**	-0.639**	-0.690**

[a] BD, soil bulk density; RWD, root weight density; RLD, root length density; RVD, root volume density; RSD, root surface area density; IAA, Indoleacetic acid; ABA, abscisic acid; TZR, trans zeatin riboside; RTR, root TTC reduction activities; RTA, root total absorption area; RAA, root active absorption area; MDA, malondialdehyde; SOD, superoxide dismutase; CAT, catalase; SP, soluble protein. [b] **P < 0.01; ns, not significant.

Table S3

Average economic cost benefits under various tillage practices in the 2013–2016 growing seasons.

Item		P[a]	R	SR	SRS
Inputs	Seed (US$ ha^{-1})	74	74	74	74
	Fertilizer (US$ ha^{-1})	375	375	375	375
	Herbicide (US$ ha^{-1})	138	138	138	138
	Irrigation cost (US$ ha^{-1})	63	63	63	82
	Mechanical operation cost (US$ ha^{-1})	780	751	686	758
	Salary (US$ ha^{-1})	266	266	266	411
	Total (US$ ha^{-1})	1696	1667	1601	1839
Outputs	Yield (kg ha^{-1})	8769	8165	7948	9816
	Price (US$ kg^{-1})	0.3148	0.3148	0.3148	0.3148
	Income (US$ ha^{-1})	2756	2567	2498	3086

[a] P, plowing tillage; R, rotary tillage; SR, strip rotary tillage; SRS, strip rotary tillage after subsoiling.

Table S4

Test of homogeneity of variance.

	RWD[a]	RLD	RVD	RSD	RTR	RTA	RAA	MDA	SOD	CAT	SP
Test of homogeneity of variances	NS[b]	NS	NS	NS	NS	NS	NS	NS	NS	NS	NS

[a] RWD, root weight density; RLD, root length density; RVD, root volume density; RSD, root surface area density; RTR, root TTC reduction activities; RTA, root total absorption area; RAA, root active absorption area; MDA, malondialdehyde; SOD, superoxide dismutase; CAT, catalase; SP, soluble protein. [b] NS, not significant.

Table S5

Correlation analysis between integrated score for root quality, grain yield and soil bulk density.

	Integrated score	Grain yield	Soil bulk density
Integrated score	1		
Grain yield	0.856**[a]	1	
Soil bulk density	-0.899**	-0.902**	1

[a] *$P < 0.05$; **$P < 0.01$.

Table S6

General description of the experimental site.

Basic information	Yanzhou
Location	35°40′ N, 116°41′ E
Above sea level (mm)	55
Climate	Warm-temperate, semi-humid continental monsoon climate
Average temperature (°C)	13.6
Annual precipitation (mm)	621.2
Accumulated sunshine hours (h)	2460.9
Groundwater depth (mm)	25
Agrotype	Loam
FAO soil classification	Haplic luvisols
Clay content (%)	29.6
Silt content (%)	37.3
Sand content (%)	33.1
pH	7.6
Cropping system	Double-cropping, maize/wheat annually
Tillage practices	Rotary or plowing tillage

Table S7

Operational procedures of various tillage practices.

Tillage	Operational procedures
Plowing tillage (P)	Returning maize straw to the field→ Spreading base fertiliser → Mouldboard plowing once with ILFQ330 turnover plough (working depth was about 25 cm)→Rotary cultivating two times with IGQN-200K-QY rotary cultivator (working depth was about 15cm)→Harrowing two times→ Forming the border-check→ Seeding with common seeder
Rotary tillage (R)	Returning maize straw to the field→ Spreading base fertiliser → Rotary cultivating two times with IGQN-200K-QY rotary cultivator (working depth was about 15 cm)→Harrowing two times → Forming the border-check→ Seeding with common seeder
Strip rotary tillage (SR)	Returning maize straw to the field→ Completing rotary cultivation of sowing row (working depth was about 15 cm), application of base fertilizer, seeding and forming border-check at the same time with the 2BMYF-10/5 multifunctional direct seeder in stubble (The row spacing of 2BMYF-10/5 multifunctional direct seeder in stubble was designed to be 9 cm + 16 cm, in which the sowing row spacing was 9 cm, so that the area of rotary cultivation took up 36 percent of the border check's total area)
Strip rotary tillage after subsoiling (SRS)	Returning maize straw to the field→ Subsoiling once with the ZS-180 vibration subsoiler (working depth was about 38 cm)→Completing rotary cultivation of sowing row (working depth was about 15 cm), application of base fertilizer, seeding and forming border-check at the same time with the 2BMYF-10/5 multifunctional direct seeder in stubble
